# Metformin exerts anti-cancerogenic effects and reverses epithelial-to-mesenchymal transition trait in primary human intrahepatic cholangiocarcinoma cells

**DOI:** 10.1038/s41598-021-81172-0

**Published:** 2021-01-28

**Authors:** Sabina Di Matteo, Lorenzo Nevi, Diletta Overi, Nadine Landolina, Jessica Faccioli, Federico Giulitti, Chiara Napoletano, Andrea Oddi, Augusto M. Marziani, Daniele Costantini, Agostino M. De Rose, Fabio Melandro, Maria C. Bragazzi, Gian Luca Grazi, Pasquale B. Berloco, Felice Giuliante, Giuseppe Donato, Lorenzo Moretta, Guido Carpino, Vincenzo Cardinale, Eugenio Gaudio, Domenico Alvaro

**Affiliations:** 1 Department of Translational and Precision Medicine, Sapienza University of Rome, Rome, Italy; 2 Department of Anatomical, Histological, Forensic Medicine and Orthopedics Sciences, Sapienza University of Rome, Rome, Italy; 3 Department of Experimental Medicine, Sapienza University of Rome, Rome, Italy; 4 Gastroenterology Unit, Regina Elena National Cancer Institute, Rome, Italy; 5 Department of Information, Electronics and Telecommunications Engineering, Sapienza University of Rome, Rome, Italy; 6 Hepatobiliary Unit, Catholic University of the Sacred Heart School of Medicine, Rome, Italy; 7 Department of General Surgery and Organ Transplantation, Sapienza University of Rome, Rome, Italy; 8 Medical-Surgical and Biotechnologies Sciences, Polo Pontino, Sapienza University of Rome, Rome, Italy; 9 Department of Movement, Human and Health Sciences, Division of Health Sciences, University of Rome “Foro Italico”, Rome, Italy; 10 Present Address: Department of Immunology, Bambino Gesù Children’s Hospital, IRCCS, Rome, Italy; 11 Present Address: Department of Biosciences, University of Milan, Milan, Italy

**Keywords:** Cancer microenvironment, Cancer prevention, Cancer stem cells, Cancer therapy, Gastrointestinal cancer, Metastasis, Cancer microenvironment, Cancer prevention, Cancer stem cells, Gastrointestinal cancer, Metastasis, Cancer, Cell biology, Gastroenterology, Oncology

## Abstract

Intrahepatic cholangiocarcinoma (iCCA) is a highly aggressive cancer with marked resistance to chemotherapeutics without therapies. The tumour microenvironment of iCCA is enriched of Cancer-Stem-Cells expressing Epithelial-to-Mesenchymal Transition (EMT) traits, being these features associated with aggressiveness and drug resistance. Treatment with the anti-diabetic drug Metformin, has been recently associated with reduced incidence of iCCA. We aimed to evaluate the anti-cancerogenic effects of Metformin in vitro and in vivo on primary cultures of human iCCA. Our results showed that Metformin inhibited cell proliferation and induced dose- and time-dependent apoptosis of iCCA. The migration and invasion of iCCA cells in an extracellular bio-matrix was also significantly reduced upon treatments. Metformin increased the AMPK and FOXO3 and induced phosphorylation of activating FOXO3 in iCCA cells. After 12 days of treatment, a marked decrease of mesenchymal and EMT genes and an increase of epithelial genes were observed. After 2 months of treatment, in order to simulate chronic administration, Cytokeratin-19 positive cells constituted the majority of cell cultures paralleled by decreased Vimentin protein expression. Subcutaneous injection of iCCA cells previously treated with Metformin, in Balb/c-nude mice failed to induce tumour development. In conclusion, Metformin reverts the mesenchymal and EMT traits in iCCA by activating AMPK-FOXO3 related pathways suggesting it might have therapeutic implications.

## Introduction

Cholangiocarcinoma (CCA) represents the second most frequent liver cancer that develops as a highly aggressive tumour with both a poor prognosis and increasing incidence worldwide. The epidemiology of CCA is continuously increasing and there are no prevention and treatment strategies to date^1^.

Intrahepatic CCA (iCCA) comprises a heterogeneous group of malignancies arising from the intrahepatic bile ducts. At histological level, iCCA shows high inter-tumoral heterogeneity; and according to the most updated nomenclature, it is classified into: Small bile duct-type iCCA and Large bile duct-type iCCA^2,3^. The first consists of a Small‐sized tubular or acinar adenocarcinoma with no or minimal mucin production; whereas the second one is constituted by mucin‐producing columnar tumour cells arranged in a large‐duct or papillary architecture^2–6^.

The histological appearance might reflect a different origin i.e.from large intrahepatic bile ducts with associated peribiliary glands (PBGs) or from interlobular bile ducts, bile ductules and canals of Hering^5^.

We have recently demonstrated that iCCA is particularly enriched of putative cancer stem cells (CSC) and of cells expressing epithelial-to-mesenchymal transition (EMT) traits^7–11^. A typical feature of iCCA is its desmoplastic nature where the mesenchymal fraction predominates over the epithelial part, with a large proportion of cancer cells expressing EMT traits.

The epithelial to mesenchymal transition (EMT) plays crucial roles in the tumour invasion process. During EMT process, the epithelial cells lose both their junctions and the apical–basal polarity and acquire features of mesenchymal cells^12^. Recent evidence indicate that EMT is a key process for tumour progression and spreading^12^ and, in the case of iCCA, EMT plays a key role in the cells increased motility, tumour progression, metastatic potential, and prognosis^13^.

The tumour microenvironment plays an important role in facilitating cancer metastasis and may induce the occurrence of EMT in tumour cells.

Apart from the microenvironment, many variables may contribute to the induction of EMT in cancer cells including inflammatory cells infiltrating the tumour site, hypoxia existing in a large area of tumour, stem cells and mesenchymal stem cells (MSCs) both present in tumour microenvironment^11,14^.

Large-scale epidemiologic studies have shown that patients treated with the anti-diabetic drug Metformin showed a reduced incidence of different cancers of the gastrointestinal tract^15–17^*.* As far as CCA is concerned, Saengboonmee et al*.*^18^ and Chaiteerakij et al*.*^19^ showed that the risk of iCCA increased 3.6-folds in diabetic patients (vs controls) and that, compared with diabetic patients not treated with Metformin, the use of Metformin was associated with a 60% reduction in iCCA risk. However, although Metformin may reduce cancer risk, an effect on survival of CCA patients was not yet demonstrated^20^. These clinical observations were corroborated by basic studies where the potential anti-cancer effect of Metformin has been investigated in different cancer cells^21–23^. These studies suggested that Metformin could act via the AMPK/mTORC1 pathway and induce the cell cycle arrest^24^. Furthermore, Metformin inhibited CCA tumour growth via the regulation of Drosha-mediated expression of multiple carcinogenic miRNAs^25^. Finally, Metformin has been demonstrated to inhibit cell signalling pathways involved in EMT and through these mechanisms to exert anti-cancer effects^26,27^. Limited information exists on the effects of Metformin on iCCA cells.

Therefore, the aim of the present study is to evaluate the effects of Metformin on primary cultures of human iCCA in terms of cell proliferation, apoptosis, invasion and migration, expression of EMT traits and cell signalling, and in vivo tumorigenicity in subcutaneous xenografts. The main result indicates that, prolonged treatment with Metformin, is able to reverse EMT traits in iCCA cells and this could represent a main mechanism of the anti-cancer effect of this drug.

## Results

### Primary iCCA cell cultures

**Figure 1:**
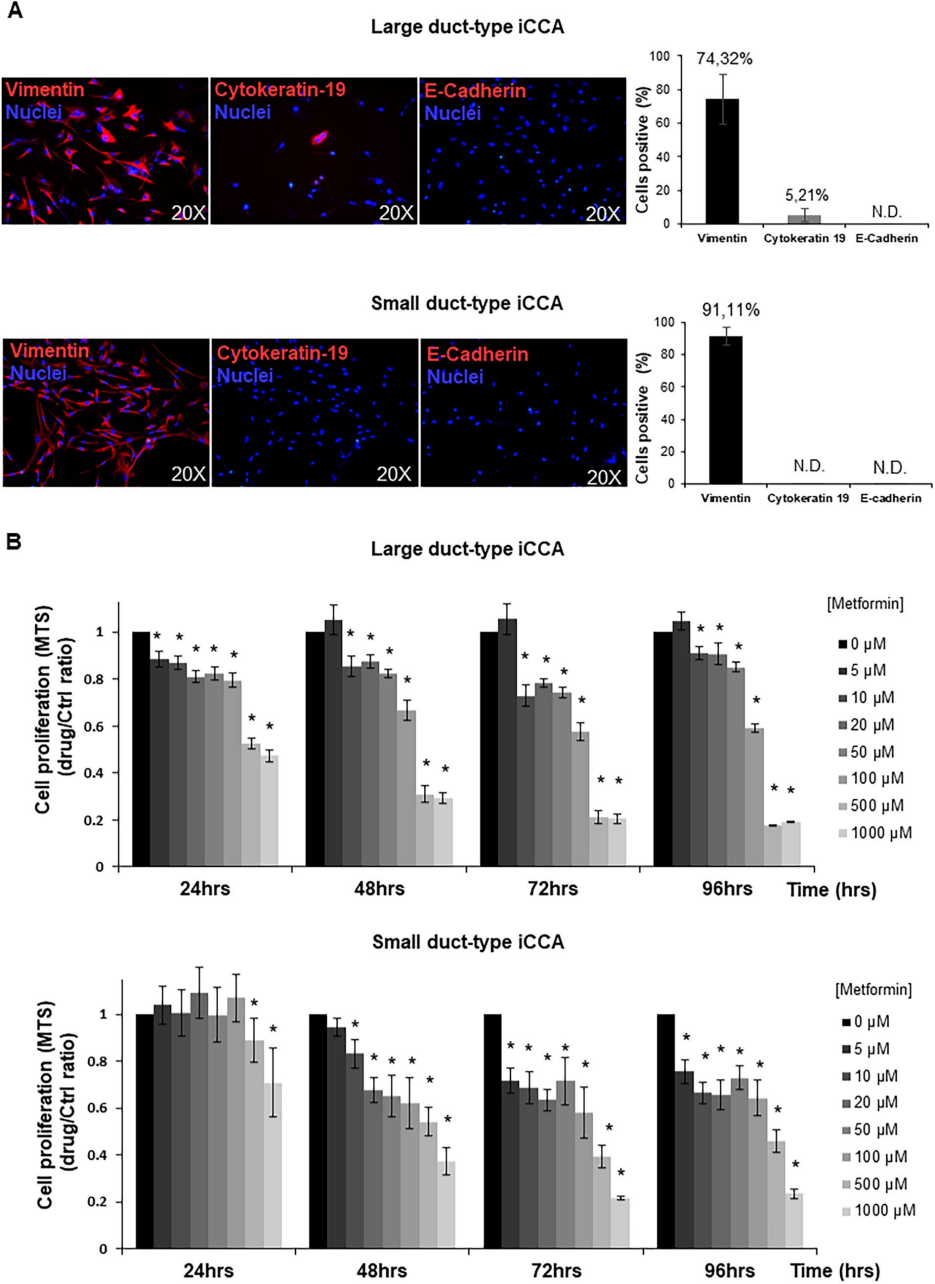
Phenotypic characterization of iCCA primary cell cultures and effect of Metformin on cell proliferation of iCCA primary cultures. (**A**) Immunofluorescence for Vimentin, Cytokeratin-19 and E-Cadherin in Large duct-type iCCA (up) and Small duct-type iCCA (down) iCCA primary cell cultures; nuclei were stained with DAPI. The bar graph indicates the percentage of positive cells. More than 70% of cells stained positive for Vimentin while express very low level of Cytokeratin-19 (< 6% of positive cells) and were negative for E-Cadherin. (See also Supplementary Figure 3A). Figures are representative of N = 5 different cell cultures. Data in the bar graph represent mean ± SD of N = 5 independent experiments. (**B**) Large duct-type iCCA (up) and Small duct-type iCCA (down) primary iCCA cell cultures were exposed for 24, 48, 72, 96 h to increasing concentration of Metformin (0–1000 µM); cell proliferation was evaluated by MTS assay and expressed as ratio with respect to controls. Metformin significantly reduced cell proliferation in a dose-dependent manner. In most experiments the inhibitory effect of Metformin on both Large and Small duct-type iCCA started at 5–10 µM. *p < 0.05 versus controls. Data represent mean ± SD of N = 5 independent experiments.

Primary iCCA cell cultures were prepared from surgical specimens of Large and Small duct-type iCCAs. Representative images of the histology and classification of tumour subtypes were included in Supplementary Figure 3B. Irrespectively to histology of the tumour of origin, iCCA cell cultures stably expressed mesenchymal and EMT markers (Fig. 1A), such as Vimentin, while on the contrary, cells expressed very low level (< 6% of cells) of the epithelial marker Cytokeratin-19 and were virtually negative for E-Cadherin (Fig. 1A) as demonstrated by immunofluorescence (IF). Primary cell cultures were negative for markers of hematopoietic cells (CD45), macrophages (CD163), activated hepatic stellate cells (GFAP), endothelial cells (CD31), fibroblast-activation protein (FAP), and stromal-derived factor (SDF1) (not shown).

### Metformin inhibited cell proliferation and induced apoptosis of iCCA cells

**Figure 2:**
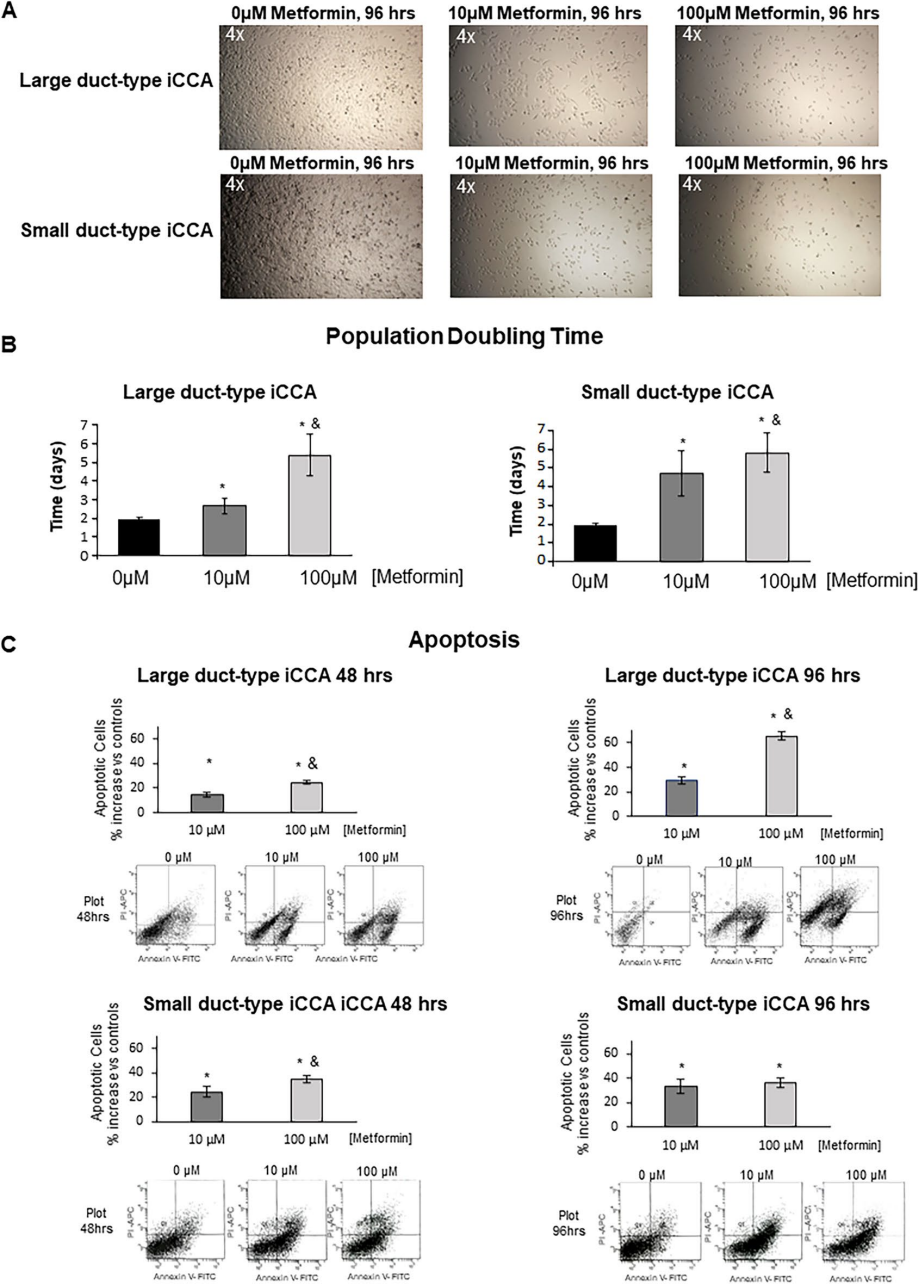
Effect of Metformin on cell density, population doubling time (PDT) and apoptosis of primary iCCA cell cultures. (**A**) Optical microscopy pictures of primary cultures of Large and Small duct-type iCCA exposed to Metformin 10 µM or 100 µM for 96 h; the decreased cell density (vs controls) is clearly evident. Data represent N = 5 independent experiments. (**B**) PDT was calculated as the time (days) required by cell cultures to duplicate their cell number. Metformin markedly elongated the PDT. Data represent mean ± SD of N = 5 independent experiments. *p < 0.05 versus controls. ^&^*p* < 0.05 versus 10 µM. (**C**) Apoptosis was assessed in both Large and Small duct-type iCCA primary cell cultures by Annexin-V/PI assay. The percentage of apoptotic cells significantly increased after treatment for 48 or 96 h with Metformin 10 µM or 100 µM. Data represent mean ± SD of N = 5 independent experiments; *p < 0.05 versus controls; ^&^*p* < 0.05 versus 10 µM.

At the optical microscopy, both in Large and Small duct-type iCCA cells, after 48 h (data not shown) and 96 h of drug exposure, the cell density is lower and the effect is greater at the concentration of 100 µM compared to 10 µM (Fig. 2A). At these doses and times, the cells are scattered, but no morphological changes ware observed in contrast to what was observed by Ling et al.^24^ on commercial CCA cells.

Primary iCCA cell cultures, exposed for 24–96 hs to increasing concentration of Metformin (0–1000 µM, Fig. 1B), showed a significant dose-dependent decrease of cell proliferation (MTS assay). The inhibitory effect of Metformin on both iCCA subtypes started to be significant (Fig. 1B) at 10 µM, corresponding to the plasmatic concentration of Metformin in patients taking the drug^28–30^. As a consequence, the population doubling time (PDT) of our primary cell cultures was significantly elongated by exposure to Metformin (Fig. 2B).

Metformin induced apoptosis of both Large and Small duct-type iCCA primary cultures as assessed by Annexin-V/PI assay (Fig. 2C). The pro-apoptotic effect of Metformin was significant after 48 h and even higher after 96 h of exposure compared to 48 h (*p* < 0.05), and at 100 µM compared to 10 µM concentration (*p* < 0.05) in Large duct-type iCCA.

### Metformin impaired cell migration and invasion of iCCA cells

**Figure 3:**
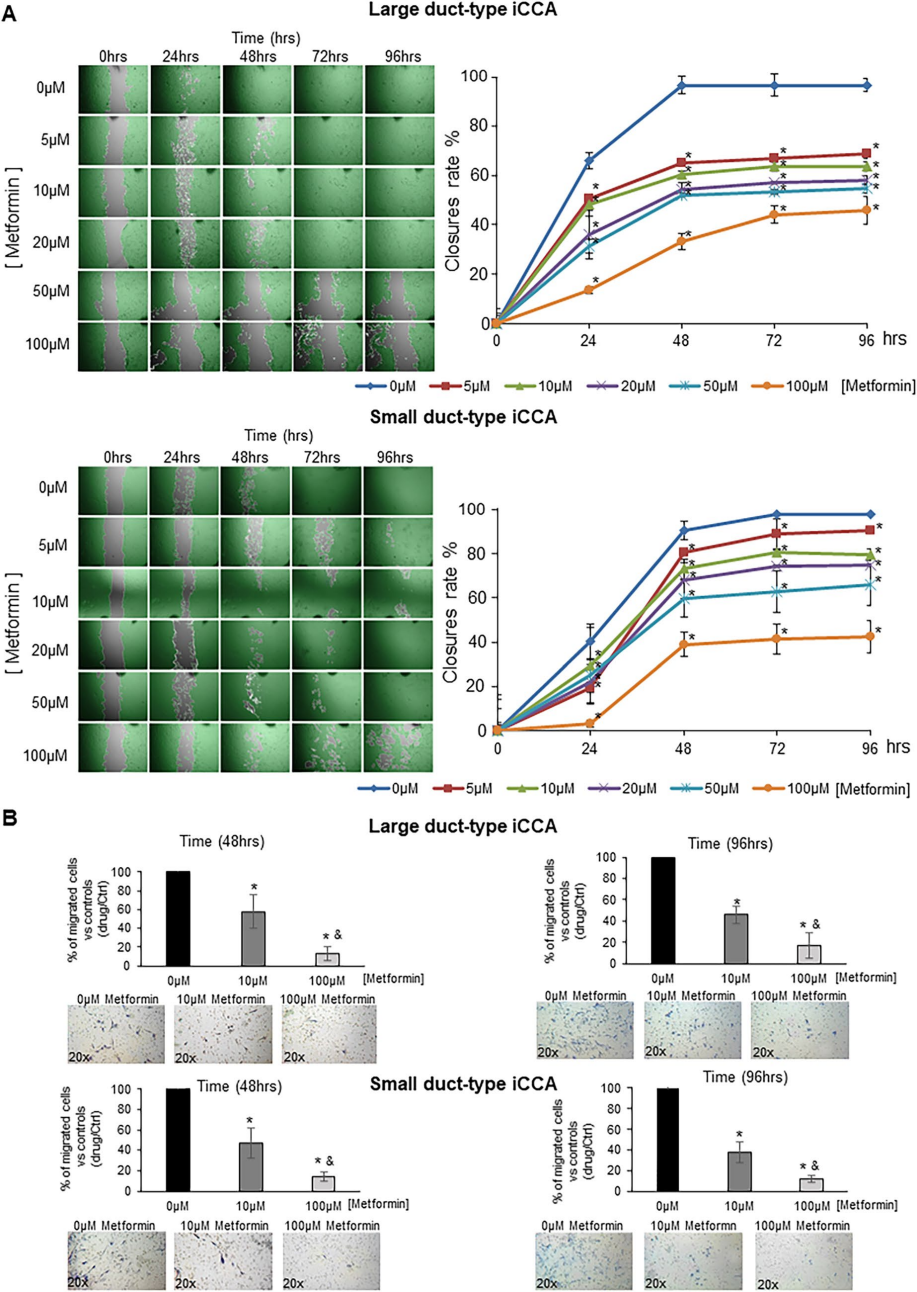
Effect of Metformin on migration and invasion of iCCA primary cell cultures. (**A**) Cell migration was analysed by the wound healing assay method in monolayer of Large duct-type iCCA or Small duct-type iCCA primary cultures treated with increasing doses (0–100 µM) of Metformin. The time required to cover the stretch (grey areas) by cell migration was significantly longer in cell cultures exposed to Metformin and this was dose-dependent. On the right, results were graphically expressed; the percentage of area covered by cell migration (after stretch) was calculated compare to controls at t_0_ (closure rate percentage). Data represent mean ± SD of N = 5 independent experiments. *p < 0.05 versus t_0_. (**B**) Cell invasion: the number of cells that migrated through the matrix were counted and the results were expressed as a percentage of migrated cells in Metformin-treated versus controls. After 48 and 96 h of treatment with Metformin 10 µM or 100 µM, the percentage of cell migrating into the matrix was significantly lower with respect to controls and this was found in both Large and Small duct-type iCCA cell cultures. Data represent mean ± SD of N = 5 independent experiments. *p < 0.05 versus controls; ^&^*p* < 0.05 versus 10 µM.

Cell migration was analysed by the wound healing assay^31^ (Fig. 3A). A scratch was performed in the monolayer of iCCA primary cultures, and wound healing was assessed after treatment with increasing doses (0–100 µM) of Metformin. Figure 3A shows that the time required to cover the scratch by cell migration was significantly longer in cell cultures exposed to Metformin (*p* < 0.05 vs controls) at 10 µM. In order to confirm that the observed changes in cell migration were not influenced by cell number changes (cell proliferation)^32,33^, an invasion assay in Matrigel (an extracellular bio-matrix) was also performed ^.^(Fig. 3B). As shown in Fig. 3B, the percentage of cells invading the membrane and the Matrigel coating was significantly decreased by Metformin starting from 10 µM concentration.

### Metformin decreased the gene expression of mesenchymal and EMT markers while increased the gene expression of epithelial markers in iCCA cells

**Figure 4:**
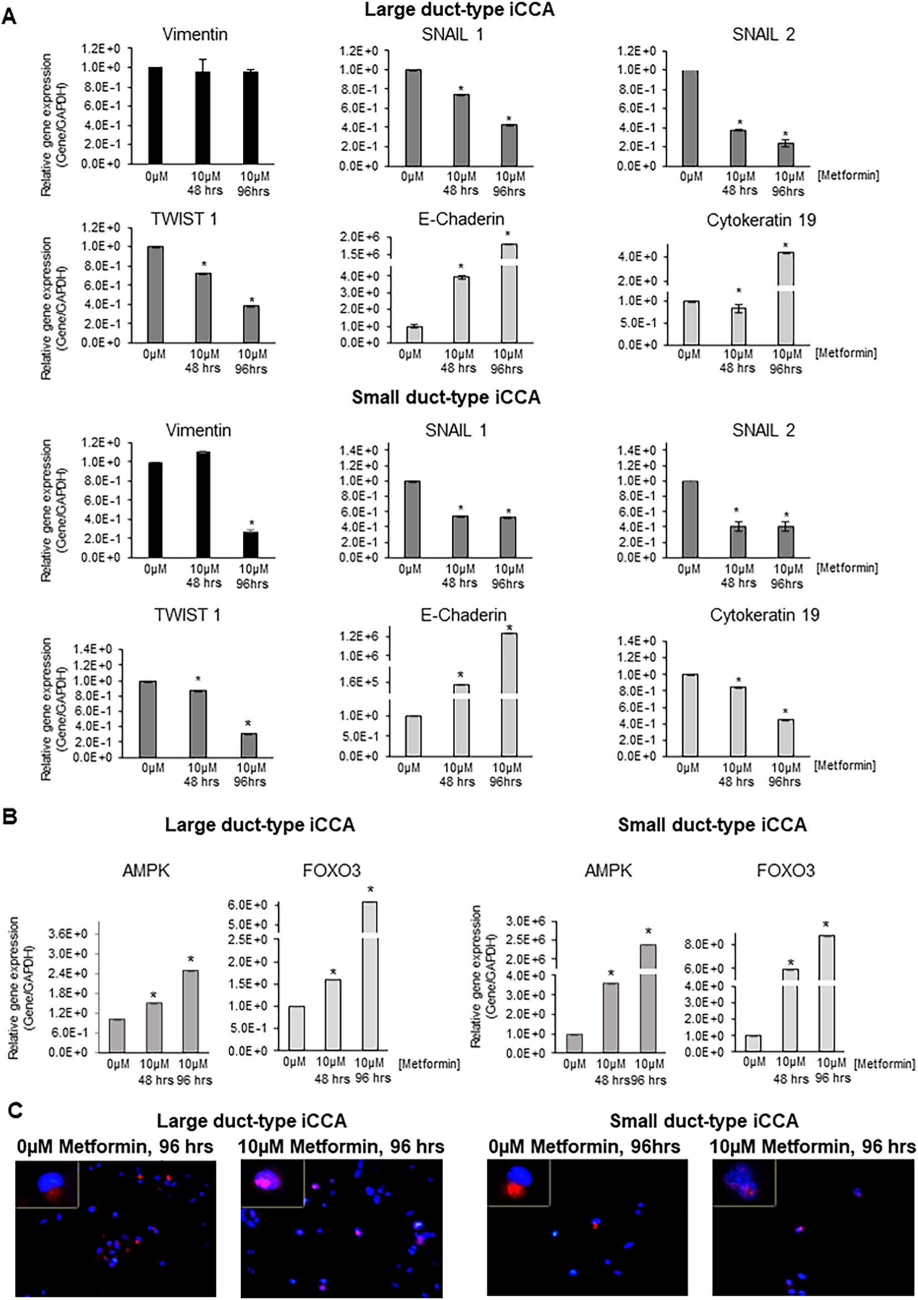
Effect of Metformin on gene expression of mesenchymal, EMT, epithelial markers, AMPK and FOXO3 in iCCA primary cells and nuclear translocation of FOXO3 in iCCA primary cell cultures. (**A**) The gene expression of mesenchymal and EMT markers (Vimentin, SNAIL1, SNAIL2, TWIST1) and epithelial markers (E-Cadherin, Cytokeratin-19) was analysed by RT-qPCR in Large and Small duct-type iCCA primary cultures exposed to Metformin 10 µM for 48 and 96 h and normalized to the expression of GAPDH (housekeeping gene). In both iCCA subtypes, the gene expression of Vimentin was significantly decreased by Metformin only after 96 h while the expression of SNAIL1, SNAIL2 and TWIST1 decreased after 48 and 96 h. In contrast, the gene expression of the epithelial marker E-Cadherin significantly increased after 48 and 96 h in both Large and Small duct-type iCCA while Cytokeratin-19 increased only in Large duct-type iCCA after 96 h of exposure to Metformin. Data represent mean ± SD of N = 5 independent experiments. *p < 0.05. (**B**) The gene expression of AMPK and FOXO3, two signalling molecules involved in the mechanism of action of Metformin, was analysed by RT-qPCR and normalized to the expression of GAPDH (housekeeping gene). After 48 and 96 h, Metformin 10 µM significantly induced the gene expression of AMPK and FOXO3 in both Large and Small duct-type iCCA primary cultures. Data represent mean ± SD of N = 5 independent experiments. *p < 0.05. (**C**) IF analysis of FOXO3 in Large and Small duct-type iCCA primary cultures exposed for 96 h to Metformin 10 µM. Migration of FOXO3 from the cytoplasm to the nucleus is evident and occurred in 64 ± 10% of the cells. Nuclei were stained with DAPI. Magnification 20 × and 10 × ; representative images of N = 3 independent experiments.

We analysed the gene expression of mesenchymal and EMT markers (Vimentin, SNAIL1, SNAIL2, TWIST1) and epithelial markers (Cadherin 1, Cytokeratin-19) in iCCA primary cultures exposed to Metformin 10 µM for 48 and 96 h (Fig. 4A) by RT-qPCR. In both iCCA subtypes, Large and Small duct-type iCCA, the gene expression of Vimentin was significantly decreased by Metformin only after 96 h while the expression of SNAIL1, SNAIL2 and TWIST1 was significantly decreased after 48 h. In contrast, the gene expression of the epithelial marker Cytokeratin-19 significantly (*p* < 0.05) increased after 48 and 96 h in both Large and Small duct-type iCCA cells while Cytokeratin-19 increased only in Large duct-type iCCA primary cell cultures and after 96 h of exposure to Metformin.

### Metformin induced the expression of FOXO3 and AMPK in iCCA cells

We analysed, in iCCA cells exposed to Metformin 10 µM , the gene expression of AMPK and FOXO3, two signalling molecules involved in the mechanism of action of Metformin by RT-qPCR^27^. As shown in Fig. 4B, treatment with Metformin 10 µM for 48 and 96 h significantly (*p* < 0.05) induced the gene expression of AMPK and FOXO3 in iCCA primary cultures. In addition, IF analysis demonstrated (Fig. 4C) the migration of FOXO3 from the cytoplasm to the nucleus in iCCA cells exposed to Metformin 10 µM for 96 h and this was indicative of FOXO3 activation.

Supplementary Table 1 showed the R Pearson correlation coefficient between the expression of AMPK or FOXO3 and the epithelial genes (E-Cadherin, Cytokeratin-19) or mesenchymal and EMT genes (Vimentin, SNAIL1, SNAI2, TWIST1) in iCCA cells exposed to Metformin. The gene expression of AMPK and FOXO3 correlated (*p* < 0.01) positively and linearly with the expression of epithelial genes (E-Cadherin, Cytokeratin-19) and negatively and linearly (*p* < 0.01) with the expression of mesenchymal and EMT genes (Vimentin, SNAIL1, SNAI2, TWIST1), suggesting a close relationship between activation of AMPK/FOXO3 and the reversal of EMT traits.

### Prolonged treatment with Metformin induced genotypic and phenotypic changes of iCCA cells suggestive of mesenchymal to epithelial transition (MET)

**Figure 5:**
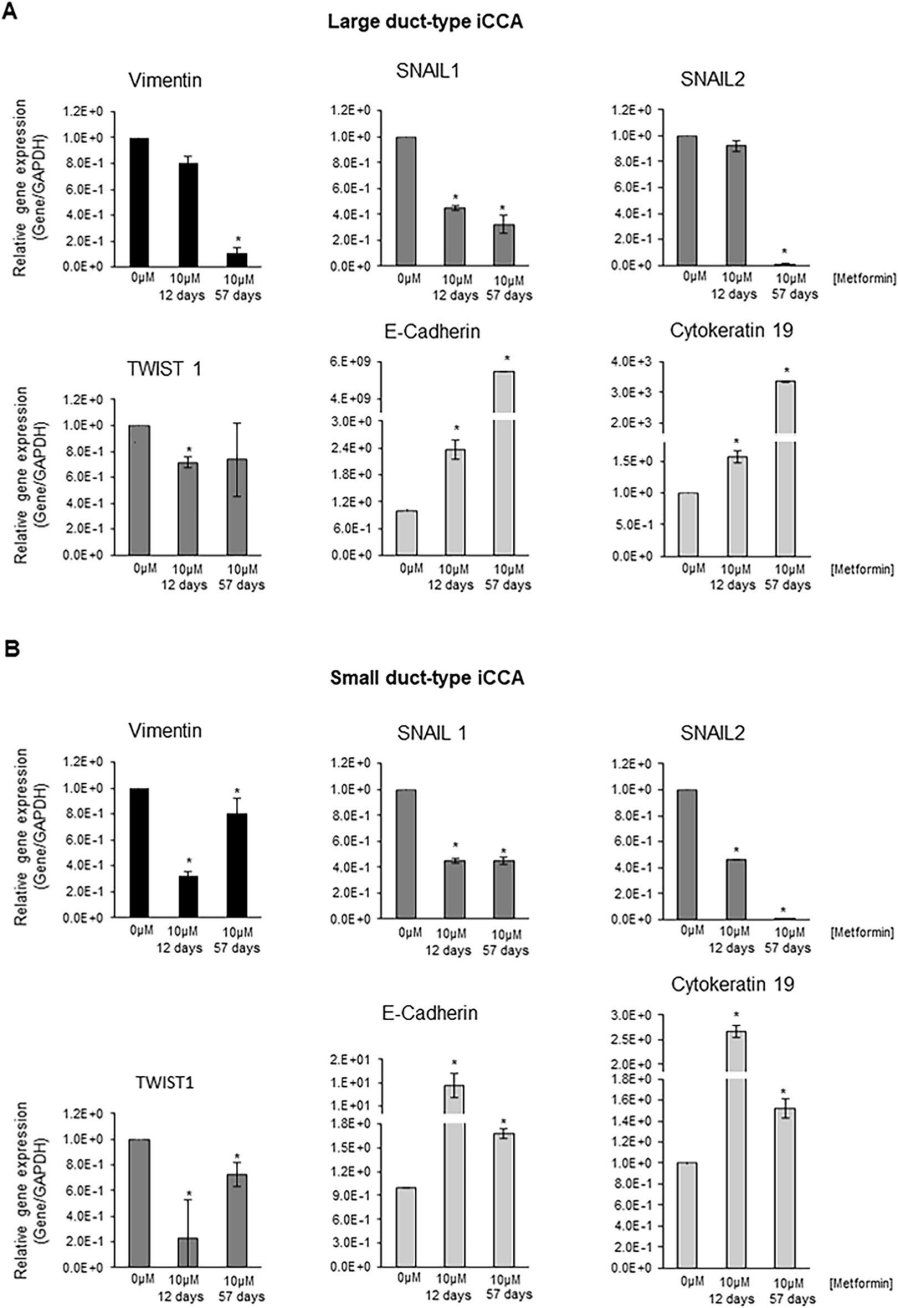
Effect of prolonged treatment (12 and 57 days) of iCCA primary cells with Metformin on the gene expression of mesenchymal, EMT and epithelial markers. The gene expression of mesenchymal and EMT markers (Vimentin, SNAIL1, SNAIL2, TWIST1) and epithelial markers (E-Cadherin, Cytokeratin-19) was analysed by RT-qPCR in Large duct-type iCCA (**A**) and Small duct-type iCCA (**B**) primary cultures exposed to Metformin 10 µM for 12 or 57 days and normalized to the expression of GAPDH (housekeeping gene). The mesenchymal and EMT markers, Vimentin, SNAIL1, SNAIL2 and TWIST1, were markedly down-regulated with respect to controls; in some case almost completely suppressed (i.e. Vimentin and SNAIL2 in Large duct-type iCCA and SNAIL2 in Small duct-type iCCA). The epithelial markers, E-Cadherin and Cytokeratin-19 were markedly upregulated in both Large and Small duct-type iCCA. Data represent mean ± SD of N = 5 independent experiments; *p < 0.05 versus controls.

**Figure 6:**
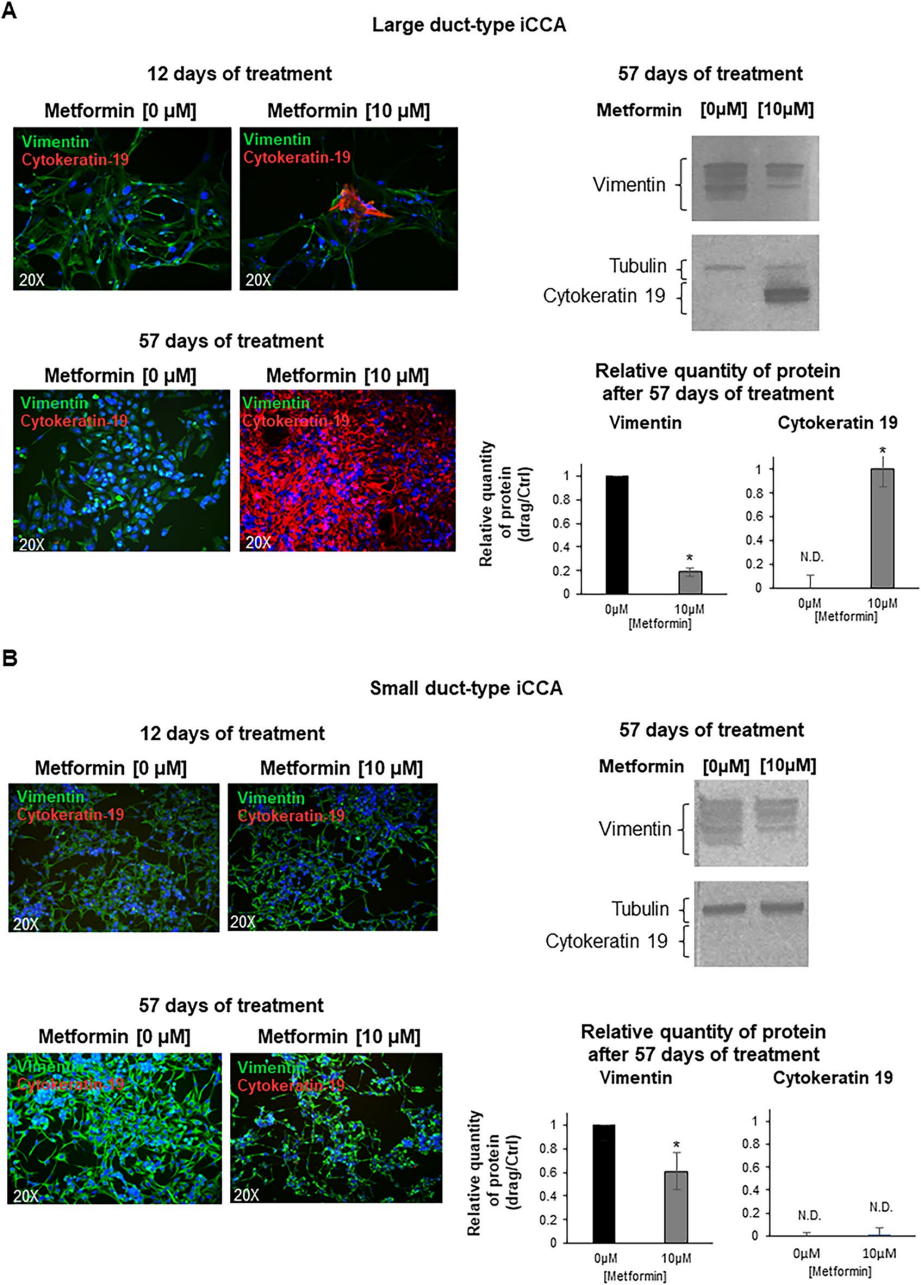
IF and western blot analysis of Vimentin and Cytokeratin-19 in primary cultures of Large duct-type iCCA treated for 12 or 57 days with Metformin. (**A**) Vimentin and Cytokeratin-19 were analysed by IF (left panels), in Large duct-type iCCA primary cultures treated with Metformin 10 µM for 12 and 57 days. Metformin induced a marked IF positivity for Cytokeratin-19 and a marked decrease of Vimentin expression that were not observed after 48–96 h of treatment. By western blot (right panels, cropped gels/blots are displayed), the protein expression of Vimentin decreased while that of Cytokeratin-19 increased after prolonged (12 and 57 days) treatment with Metformin 10 µM. *p < 0.05 versus controls. Data represent mean ± SD of N = 3 independent experiments. (**B**) In contrast with Large duct-type iCCA, in the Small duct-type iCCA cells the Cytokeratin-19 expression was not observed neither by IF not by western blot (not detectable, ND). However, by western blot analysis a decreased level of Vimentin was observed. *p < 0.05 versus controls. Mean ± SD of N = 3 independent experiments.

**Figure 7:**
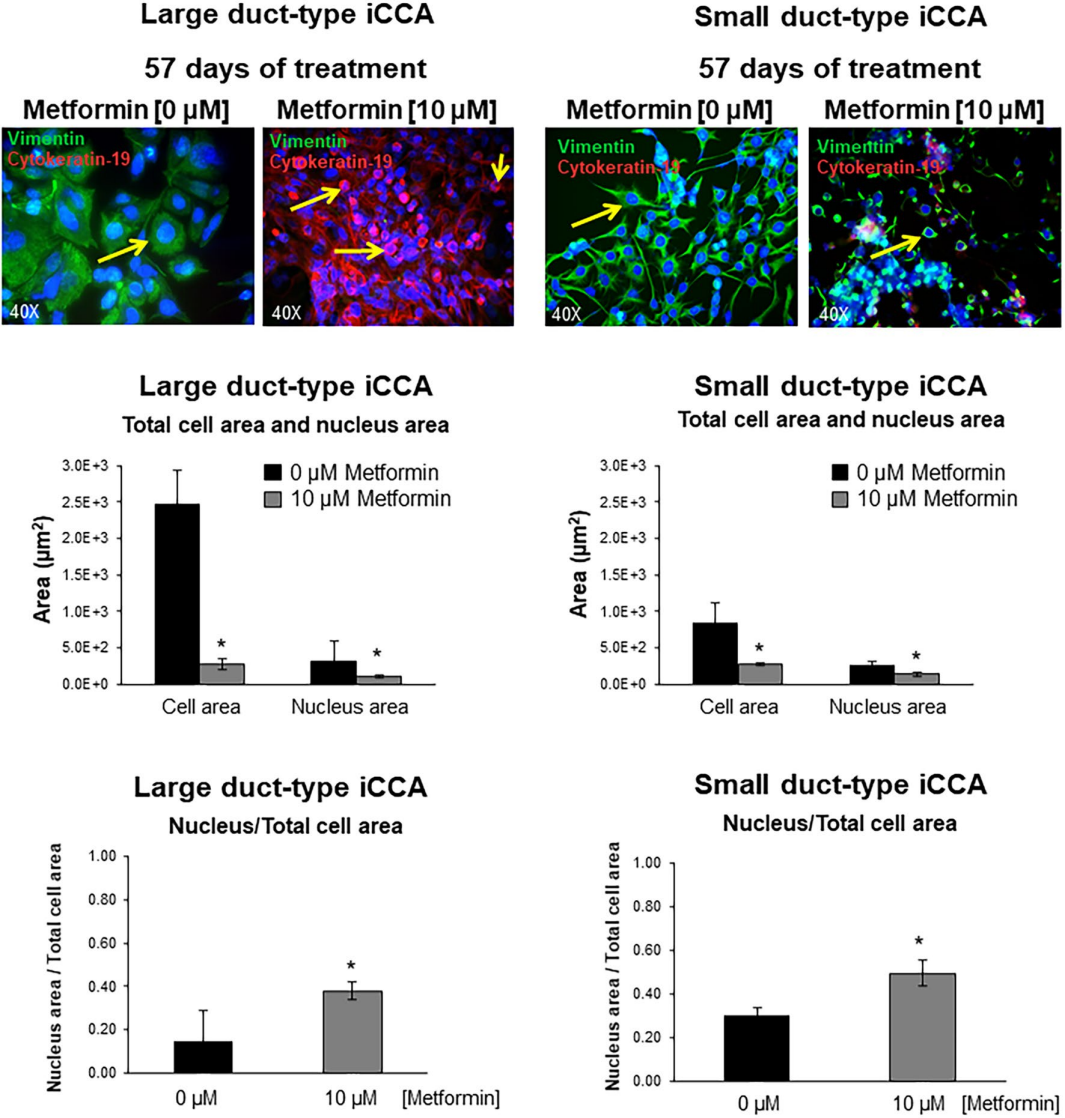
Changes of cell morphology after exposure to Metformin 10 µM for 57 days was observed in Large and Small duct-type iCCA by IF analysis. In both Large and Small duct-type iCCA, significant changes of cell morphology after exposure to Metformin 10 µM for 57 days, was observed. Indeed, treated cells displayed smaller nuclei, smaller total cell area and a smaller nucleus area / total cell area ratio compare to controls; these changes being consistent with a trend toward Mesenchymal-Epithelial transition^34^. *p < 0.05 versus controls. Data represent mean ± SD of N = 3 independent experiments.

At a gene level (RT-qPCR), the mesenchymal and EMT markers, Vimentin, SNAIL1, SNAIL2, TWIST1, were markedly down-regulated by long-term (57 days, Fig. 5) versus short-term (48–96 h, Fig. 4) exposure to Metformin and, in some case, almost completely suppressed (i.e. Vimentin and SNAIL2). In contrast, the epithelial markers E-Cadherin and Cytokeratin-19 were markedly upregulated (Fig. 5A,B). Consistent with the analysis of gene expression, Large duct-type iCCA cells showed, after 57 days of treatment with Metformin 10 µM, a marked positivity for Cytokeratin-19, by IF, that was not observed after 48–96 h (data not show) (Fig. 6A). Western blot analysis confirmed the huge increase of Cytokeratin-19 and the decreased Vimentin levels after prolonged treatment with Metformin of Large duct-type iCCA cells (Fig. 6A). As evident in Fig. 7, the cell morphology changed after prolonged treatment with Metformin. Indeed, treated cells displayed smaller nuclei, total cell area and a smaller nucleus / total cell area ratio. These changes were consistent with a trend toward mesenchymal-epithelial transition^34^.

In contrast with Large duct-type iCCA, in the Small duct-type iCCA cells, we failed to observe Cytokeratin-19 enhanced expression by IF, but only decreased levels of Vimentin by western blot analysis (Fig. 6B), a decrease of cell and nucleus area and an increase of nucleus/cytoplasm area ratio (Fig. 7).

### Clonogenic assay: Metformin impaired the colony formation capacity of iCCA cells

Large and Small duct-type iCCA primary cell cultures, treated for 12 and 57 days with Metformin 10 µM, were seeded for an in vitro clonogenicity assay and showed a drastic reduction of grafting and colony formation capacity (*p* < 0.05, Supplementary Figure 1).

In Large duct-type iCCA pre-treated with Metformin the limited number of formed colonies showed a reduced size and a phenotypic change (Supplementary Figure 1A). The number of colonies of Large duct-type iCCA was: 81 ± 5 for controls (12 days, 0 µM Metformin) and 14 ± 3 for cells pre-treated for 12 days with Metformin 10 µM (*p* < 0.05 0 µM vs 10 µM). After 57 days of pre-treatment, the number of colonies for Large duct-type iCCA was 82 ± 10 for controls and 6 ± 5 for cells pre-treatment with Metformin 10 µM (*p* < 0.05 0 µM vs 10 µM). For the colony dimension, the percentage of the control cells was set at 100%. The colony dimension percentage of 12-day Metformin pre-treated-Large duct-type iCCA was: 29.3 ± 7.6% (*p* < 0.05 0 µM vs 10 µM), whereas, after 57 days of pre-treatment with Metformin 10 µM, it was 7.1 ± 6.4% (*p* < 0.05 0 µM vs 10 µM). Indeed, the margins of the untreated colonies were less defined as compared to the treated ones. The shape of the Large duct-type iCCA within the pre-treated colonies with Metformin 10 µM were less elongated and more squared. In contrast, treatment of Small duct-type iCCA with Metformin, resulted in a mild reduction of grafting capacity and colony formation capacity (Supplementary Figure 1B). After 12 days of pre-treatment, the number of colonies of Small duct-type iCCA was: 85 ± 7 for controls (Metformin 0 µM) and 50 ± 9 for cells pre-treated with Metformin 10 µM (*p* < 0.05 0 µM vs 10 µM). After 57 days of pre-treatment, the number of colonies was 85 ± 10 for controls and 32 ± 11 for cells pre-treated with Metformin 10 µM (*p* < 0.05 0 µM vs 10 µM). Metformin-treated Small duct-type iCCA colony appeared smaller compared to controls. Indeed for the colony dimension percentage the control was set at 100% whereas for pre-treated Small duct-type iCCA it was: 37.4 ± 21.8% after 12 days of pre-treatment with Metformin 10 µM (*p* < 0.05 0 µM vs 10 µM) and 38.6 ± 15.8% after 57 days of pre-treatment with Metformin 10 µM (*p* < 0.05 0 µM vs 10 µM) (Supplementary Figure 1B). However Small duct-type iCCA colony dimension percentage was higher than Large duct-type iCCA at the same time point of treatment.

### Xenografts in balb/c nude mice: Metformin prevented tumour formation in vivo

**Figure 8:**
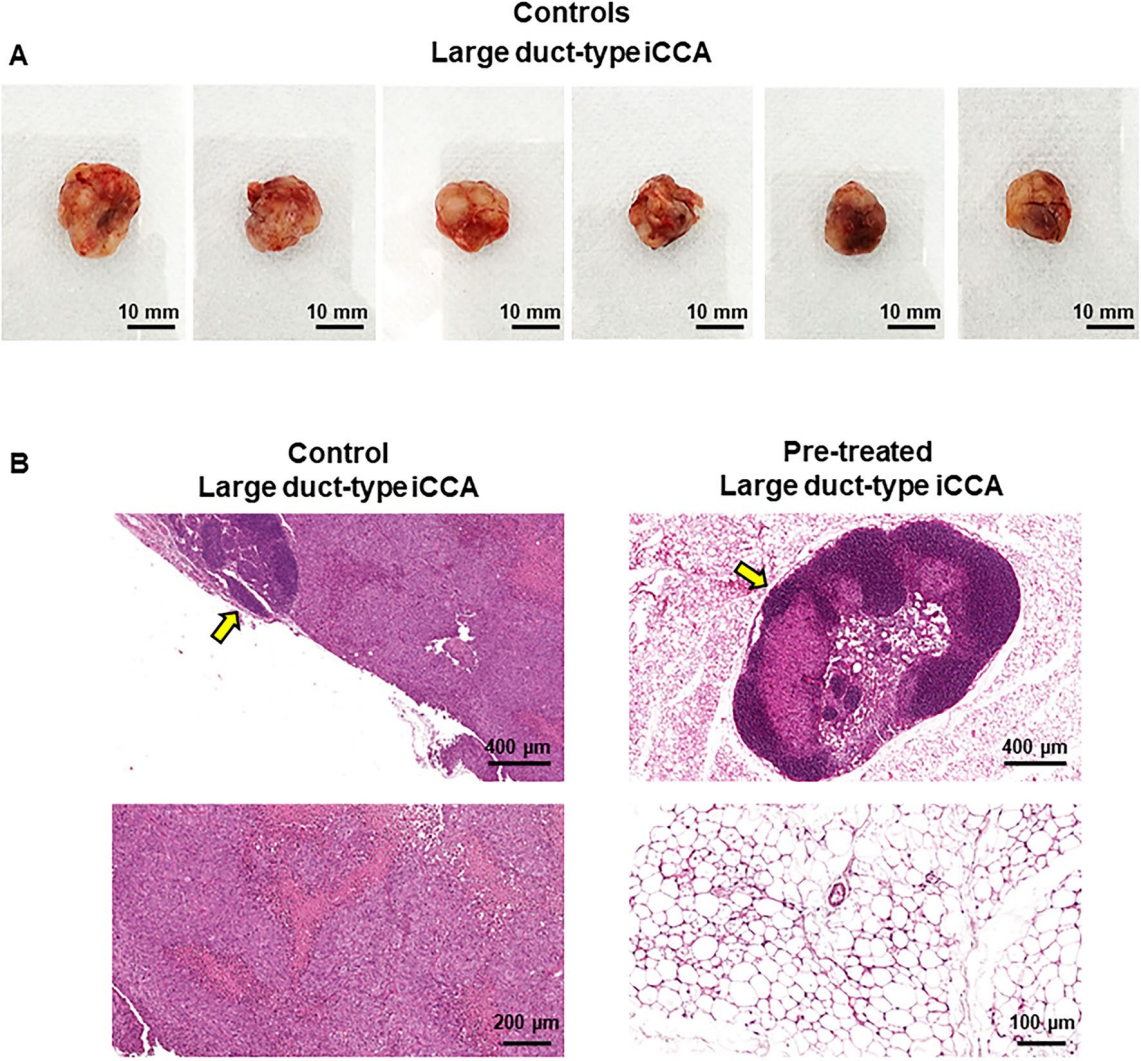
In vivo tumorigenity: xenografts in balb/c nude mice. (**A**) Photographs of Large duct-type iCCA macroscopic tumour masses removed from the flank of mice 10 weeks after the injection of Large duct-type iCCA cells. Mice injected with pre-treated Large duct-type iCCA with Metformin 10 µM for 57 days (Pre-treated) did not developed a macroscopically evident tumour mass within subcutaneous tissues at the level of the injection point while mice injected with pre-treated Large duct-type iCCA with Metformin 0 µM for 57 days (Controls) cells did. See also supplementary Figure 4. (**B**) H&E stains on explanted tissues confirm the neoplastic nature of the subcutaneous mass in mice injected with Control Large duct-type iCCA (Control, panels on the left). Neoplastic cells infiltrate subcutaneous tissues including the inguinal lymph node (arrow). In mice injected with Pre-treated Large duct-type iCCA (panels on the right), subcutaneous tissue were collected from the injection point, using inguinal lymph nodes as reference. The microscopical examination of inguinal lymph node (arrow, upper panel) and subcutaneous tissues (lower panel) indicates the absence of tumour formation at microscopical level. Representative images for N = 6 independent experiments for mice injected with pre-treated Large duct-type iCCA with Metformin 10 µM for 57 days and N = 6 for iCCA untreated cells (Metformin 0 µM, Controls).

Large duct-type iCCA primary cell cultures were treated for 57 days with Metformin 10 µM and, thereafter, injected subcutaneously in male balb/c nude mice. Controls mice were injected with untreated Large duct-type iCCA cells. After two months, a tumour mass was macroscopically evident at the flank of injection in controls mice (N = 6; Fig. 8 and Supplementary Figure 4); the mean volume of the masses was 2700 ± 100 mm^3^. The microscopical examination confirmed the neoplastic nature of the subcutaneous mass. Neoplastic cells infiltrate subcutaneous tissue including the inguinal lymph nodes. Differently, in mice in which Metformin-treated cells were injected, no mass was macroscopically appreciated at the site of injection (Supplementary Figure 4). The subcutaneous tissues at the site of injection were resected and analysed; the microscopic examination of subcutaneous tissue and inguinal lymph nodes indicated the absence of neoplastic cells.

## Discussion

The main findings of our study indicate that, in primary cultures of human Large and Small duct-type iCCA, Metformin: (1) inhibited cell proliferation and induced apoptosis in a dose- and a time-dependent manner; (2) inhibited cell migration and invasion and colony formation capacity of iCCA cells; (3) downregulated the gene expression of mesenchymal and EMT genes and upregulated epithelial genes; (4) activated AMPK and FOXO3; (5) after prolonged exposure, induced MET in Large duct-type iCCA as demonstrated by the expression of Cytokeratin-19, the down-regulation of Vimentin and changes of cell morphology; (6) abolished the in vivo capacity of Large duct-type iCCA to develop tumour after subcutaneous injection in mice. Importantly, all these findings were obtained at a concentration corresponding to the plasma level of Metformin in treated diabetic patients^28–30^. Taken together, these results indicate that Metformin could exert anti-tumoral effects on human iCCA cells.

The study was carried out in primary cultures of iCCA as obtained from specimens of surgically-resected patients. iCCA is a desmoplastic tumour characterized by tumour microenvironment with abundant stroma and composed of epithelial cells expressing EMT traits and stem cell markers^5,7^. These features were also present in the primary iCCA cultures, where almost all cells progressively lost epithelial antigens and acquired mesenchymal and EMT markers^7^. One of the most interesting results from our study is that Metformin, after prolonged treatment, may reverse the phenotype of Large duct-type iCCAs primary cultures with re-expression of Cytokeratin-19, marked downregulation of Vimentin and changes of cell morphology consistently with an epithelial phenotype, explained by a Metformin reversion of EMT.

The observed difference in the gene and protein expression profiles, in the cell shape and morphology of colonies in Small duct-type iCCA as compared to the Large duct-type iCCA might be due to a partial reversion of EMT that possibly took place in the Large duct-type iCCA but not in Small duct-type iCCA. Such differences might be correlated to distinct cell of origins, giving rise to cancer stem cells featuring the distinct iCCA subtypes that are, in turn, responsible for the generation of EMT with diverse properties, and with a differential responsiveness to drugs^2,3,11,13^.

Our results are in line with previous in vivo and in vitro studies on various cancer cell types. In these studies, indeed, it was demonstrated that Metformin exerted anti-tumoral effects by: (1) activating AMPK that plays a role in cellular energy homeostasis^35–37^, (2) inhibiting adenosine deaminase that converts AMP into IMP, resulting in AMP accumulation^38^ with a subsequent activation of AMPK^38,39^, (3) blocking the mitochondrial respiratory chain complex (NADH dehydrogenase) that impairs ATP synthesis and increasing the AMP/ATP ratio^40,41^. In addition, several studies indicated that Metformin counteracts the EMT process^42–45^, but this had never been demonstrated in CCA cells.

Recently, Chou et al*.*^46^ demonstrated the relationship between AMPK and AKT-MDM2-FOXO3 pathways, which are involved in the inhibition of the EMT process in human epithelial cells of both mammary gland and prostate^46^. In mammary glands and prostate cell lines, Metformin resulted in a positive modulation of AMPK, FOXO3 and E-Cadherin and in the inhibition of Vimentin and EMT transcription factors. In ovarian cancer stem cells, Zhang et al.^35^ showed that Metformin reduced the gene expression of Vimentin, SNAIL2 and TWIST1 and these findings were confirmed in vivo in a murine model^35^. As far as CCA is concerned, Metformin significantly suppressed proliferation of RBE and HCCC-9810 CCA cell lines in a dose- and time-dependent manner by targeting the AMPK/mTORC1 pathway^24^. Furthermore, Metformin also acts by regulating the Drosha-mediated expression of multiple carcinogenic miRNAs in HCCC-9810, RBE and SSP25 CCA cell lines^25^.

Our data on iCCA cells are consistent with this background, since we have demonstrated that Metformin stimulated AMPK and FOXO3 gene expression, the migration of the latter from the cytoplasm to nucleus, and the significant correlations between AMPK, FOXO3 and the epithelial (Cytokeratin-19 and E-Cadherin), mesenchymal (Vimentin) and EMT (SNAIL1, SNAIL2 and TWIST1) genes.

According with our findings, Saengboonmee et al*.*^47^ showed that Metformin increased anoikis and inhibited migration/invasion of CCA cell lines (KKU-055, KKU-100, KKU-213 and KKU-214) in association with decreased Vimentin expression and matrix metalloproteinase (MMP)-2 and -7 activities; activation of AMPK phosphorylation together with suppression of nuclear translocation of signal transducer and activator of transcription 3 (STAT3) and nuclear factor-kappa B (NF-ĸB) being the mechanisms involved in these effects^47,48^.

Our data suggest that also in primary cultures of human iCCA, Metformin triggered AMPK and FOXO3 gene expression; these could represent the molecular mechanisms at the basis of the cell proliferation blockage, the induction of apoptosis, and the reversion of EMT that we observed in our in vitro study (Supplementary Figure 2). Other than dose-dependent, the in vitro effect of Metformin was time-dependent. Indeed, after 48–96 h of treatment, we observed only up-regulation of epithelial genes and down-regulation of mesenchymal and EMT but, only after 57 days, we were able to demonstrate the re-expression of Cytokeratin-19 and changes of cell morphology consistent with an epithelial phenotype. While previous studies used millimolar concentrations doses of Metformin in immortalized cell line, our results were obtained in primary cultures of human iCCA, using micromolar doses that are comparable with the therapeutic target concentration in diabetic patients^24,48,49^. This further highlight potential translational opportunities of this work.

On the basis of our results and previous literature^48–52^, and considering its good safety profile in diabetic patients^53–55^ and in animal models^50,56,57^, Metformin could be tested in primary prevention of iCCA in high risk populations and might be considered for future clinical studies in association with chemotherapeutics or target agents for iCCA^45,58,59^ as already investigated for other types of cancer (NCT03359681, NCT02672488, NCT03238495, NCT02949700, NCT03477162, NCT02035787).

The most relevant risk factors for iCCA are liver flukes in eastern countries^60^ and PSC^5^ and diabetes^61^ in western countries. In these pathologic conditions, activation and proliferation of stem/progenitor cells in canals of Hering or PBGs have been considered as an early step of the repair/regeneration processes^62^. When the niches of stem/progenitor cells are chronically activated and proliferate in a milieu of inflammation, they undergo EMT. All these conditions favouring neoplastic transformation are counteracted by Metformin and support the rationale for testing Metformin in the primary prevention of iCCA. Interestingly, the use of Metformin seems to be safe in patients with cirrhosis, another risk factor for iCCA and also in this pathologic condition it could provide a survival benefit.

## Materials and methods

### Human iCCA primary cell cultures and tissue sourcing

Large and Small duct-type primary iCCA cells cultures were isolated from specimens obtained from patients presenting a single mass lesion within the liver. The patients were submitted to curative surgical resection at the ‘‘Paride Stefanini’’ Department of General Surgery and Organ Transplantation, Sapienza University of Rome, Rome, Italy; or at the Surgery, Hepatobiliary Unit, Catholic University of the Sacred Heart School of Medicine, Rome, Italy; or at the Hepato-Biliary Surgery, Regina Elena National Cancer Institute, Rome, Italy^5,6^.

The use of human materials and the research protocols were approved by our local Institutional Review Board and by the Ethics Committees of the Policlinico Umberto-I, University Hospital (Prot. 241/19 Ref. 4492) respectively.

All the procedures were in accordance with Good Manufacturing Practice (cGMP). The research protocols were reviewed and approved by the Ethic Committees of Policlinico Umberto I, Catholic University of the Sacred Heart School of Medicine and Regina Elena National Cancer Institute, Rome, Italy. Subjects have been properly instructed and have indicated that they consent to participate by signing the appropriate informed consent paperwork. No organs or tissues were procured from prisoners. No donor organs were obtained from executed prisoners or other institutionalised individuals.

The histology of the tumours was characterized by routine histomorphologic and immunohistochemistry stains as Haematoxylin and eosin (H&E), Periodic acid–Schiff (PAS) and immunohistochemistry for Cytokeratin-7 (M7018, Dako, Glostrup, Denmark). iCCA histological subtypes were classified according to established international criteria^2,3^.

Primary cell cultures were prepared by mechanical and enzymatic dissociation of specimens of human iCCA samples and cultures were maintained in H69, a hormonally supplemented medium, described in a previous work^8^ in a humidified atmosphere of 5% CO_2_ in air.

In the present study, iCCA primary cell cultures were characterized and analysed for short-term treatment with Metformin (48 h and 96 h) before the 10^th^ passage from the isolation, whereas all the results of the long-term (12 days and 57 days) experiments were obtained before the 20th passage from isolation.

### Metformin

Metformin hydrochloride (CAS Number 1115-70-4; Sigma-Aldrich, Milan, Italy) was dissolved in H69 medium as stock solution and then added in the cell culture media at a final concentration of 5, 10, 20, 50, 100, 500, or 1000 µM. After 24 h from cell seeding, the H69 culture medium was replaced by fresh medium containing or not containing (Metformin 0 µM, Controls) the drug and was changed every two days.

### Immunofluorescence

Primary cell cultures were fixed in 1:1 Acetone/Methanol (10 min at room temperature), incubated in 20% fetal bovine serum (FBS) in Dulbecco’s Phosphate Buffered Saline (DPBS) for 30 min, and then incubated for 1 h at room temperature with primary antibodies anti Human Vimentin RV202 (sc-32322, Santa Cruz Biotechnology Dallas, Texas, USA), Human Cytokeratin-19 (sc-6278, Santa Cruz Biotechnology), Human Vimentin (AlexaFluor 488 conjugated, goat IgG, IC8104G, R&D System, Minneapolis, MN, USA), Human Cytokeratin-19 (AlexaFluor 594 clone A53-B/A2, 628,504, Biolegend San Diego, CA, USA). The following secondary antibodies were used for un-conjugated primary antibodies: goat anti-Mouse IgG1 Texas Red (sc-2979, Santa Cruz Biotechnology) and goat Anti-Mouse IgG2a Texas Red (sc-2980, Santa Cruz Biotechnology); secondary antibodies were incubated for 1 h at room temperature.

For IF analysis of E-Cadherin (also known as CDH1 or Cadherin-1) cell cultures were fixed in 1:1 Methanol for 20 min at room temperature, permeabilization of cell membranes was performed with 0.25% Saponin in DPBS for 20 min and blocked in 20% FBS in DPBS for 1 h and then exposed for 1 h at room temperature to mouse anti-Human E-Cadherin (sc-21791, Santa Cruz Biotechnology) and Goat anti-Mouse IgG1 Texas Red (sc-2979, Santa Cruz Biotechnology).

For IF analysis of FOXO3, primary cell cultures were fixed in formalin 4% in DPBS (10 min at room temperature) and blocked 30 min in glycine 1 M and 1 h in 20% FBS DPBS; permeabilization of cell membranes was obtained with 1% bovine serum albumin (BSA) and 0.25% Triton X-100 in DPBS for 10 min and then cells were exposed for 1 h at room temperature to antibody mouse anti human FOXO3 (sc-48348 Santa Cruz Biotechnology). Cells were then incubated with goat anti-Mouse IgG1 Texas Red (sc-2979, Santa Cruz Biotechnology), for 1 h at room temperature.

Nuclei were stained with 0.2 µg/ml di 4,6-diamidino-2-phenylindole (DAPI, Sigma) for 2 min at room temperature.

The image acquisition was performed with Leica DM2000 Fluorescence microscope (Leica Microsystems, Milan, Italy) equipped with Leica DFC450 C digital camera (Leica Microsystems). Positive cells for the markers were counted and reported as a percentage of positive cells in the bar chart. Total cell area and nucleus area of cells were quantified through ImageJ software and reported as area in µm^2^ and as nucleus/total cell area ratio.

### RT-qPCR

Approximately 8⋅10^4^ cells / well iCCA primary cells were cultured in 6 multi-well plates with Metformin (10 µM and 100 µM) or without Metformin (Metformin 0 µM, Controls) for the indicated time points (48 h, 96 h, 12 day or 57 day). Subsequently, samples were collected and placed in TRIzol (15,596,026, Thermo Fisher Scientific inc., Waltham, MA, USA) as described in previous works^10^. The primers sequences used in the study are shown in Supplementary Table 2.

All expression levels of the genes of interest were normalized for GAPDH (Housekeeping Gene) and the results ware expressed as the relative gene expression (Gene/GAPDH).

### Western blot

Total protein extraction was obtained by adding RIPA buffer, phosphatase inhibitor cocktail-2 (1:100) (P5726, Sigma) and protease inhibitor cocktail (P8340, Sigma) (1:100) to iCCA primary cell cultures. The total protein extract was subjected to 4–20% SDS-PAGE (Mini-PROTEAN TGX BIO-RAD). The resolved proteins were transferred to a 0.2 µm pore–size nitrocellulose membrane (BIO-RAD). After blocking, each antibody was added for 1 h. The following monoclonal antibodies were used: anti-Vimentin (1:200, sc-32322); mouse monoclonal anti-Cytokeratin-19 (1:200, sc-6278); mouse monoclonal anti-Tubulin (1:1000, sc-8035) all purchased from Santa Cruz Biotechnology.

The densitometric quantization of the Western Blot bands was conducted through the ImageJ software. Gene bands was normalized to Housekeeping Gene (Tubulin) and expressed as: relative quantity of protein (drug/Control).

### Proliferation assay

Proliferation was evaluated by MTS assay (CellTiter 96 AQueous MTS Reagent Powder, Promega). Approximately a total of 8⋅10^3^ cells was seeded into 96-well plates in 100µL of culture medium. The method has been described in detail in a previous work^8^. Results were expressed as % changes compare to controls considered equal to 1.

### Population doubling time

The time required by cell cultures to duplicate their cell number (Population Doubling Time, PDT) was calculated as described by Nevi et al.^63^.

### Apoptosis assays

Approximately 8⋅10^4^ cells / well were cultured in 6 multi-well plates. After 24 h, the medium was replaced with fresh medium containing or not (Controls) the drug. Apoptosis was measured by staining with BD Pharmingen kit (556,547, BD Biosciences, San Diego, CA, USA) including Annexin-V-FITC used in conjunction with a vital dye propidium iodide (PI) to identify late apoptotic cells (Annexin-V-FITC and PI positive). The Cells were analysed by a BD FACS Canto Flow Cytometer (Becton, Dickinson and Company, NJ, USA). Ten thousand events were acquired and analysed by BD FACSDiva software (Becton, Dickinson Company, NJ, USA). Results were expressed as percentage increase of apoptotic cells compare to Controls (Metformin 0 µM).

### Migration assay and invasion assay

The evaluation of cell migration was performed by Wound Healing Assay as previous described^10^. Results were expressed as closures rate percentage of covered area by cell migration at different times (0–96 h) after treatment with increasing concentration of Metformin impaired to covered area by cell at time t_0_.

Further evaluation of in vitro cells invasion was performed using specific plates (Corning Matrigel Invasion Chamber 6-Well Plate 8.0 Micron) in accordance with the manufacturer's specifications and as already described^10^. The total migrated cells were evaluated by computing the number of cells that migrated through the matrix and the results were expressed as a percentage of cells migrated compared to controls.

### In vivo tumorigenicity: xenografts in balb/c nude mice

Human Large duct-type iCCA cells from primary cultures were treated with (Metformin 10 µM) or without (Metformin 0 µM, Controls) for 57 days. Afterwards, cells were subcutaneously injected (10^6^ cells in 100 µl Matrigel HyStem-C Cell Culture Scaffold, Sigma-Aldrich) into male BALB/c nude mice, 4–5 weeks old (mean body weight = 25 g, purchased from Charles River Laboratories, Wilmington, MA). Mice were maintained under standard conditions, in a temperature-controlled environment (20–22 °C) under 12 h light–dark cycles, according to the institutional guidelines for animal care.

The use of animals was approved by the Italian Ministry of Health according to Legislative Decree n°26/2014 on the protection of animals used for scientific purposes.

Tumour xenograft formation was evaluated by macroscopic inspection. In control mice, untreated Human Large duct-type iCCA cells from primary cultures (Metformin 0 µM, Controls) for the same time point (57 days) were injected.

After 10 weeks, mice were sacrificed by cervical dislocation, tumours were then removed and fixed in formaldehyde solution for routine histology^7,8^. Briefly, specimens were fixed in 10% buffered formalin, embedded in low-temperature–fusion paraffin and cut into 3- to 4 μm sections. Sections were stained with H&E and examined in a coded fashion by Milan, Italy Microsystems DM4500B Light Microscopy (Weltzlar, Germany), equipped with a Jenoptik Prog Res C10 Plus Videocam (Jena, Germany). Slides were scanned by a digital scanner (Aperio Scanscope CS System, Aperio Digital Pathology, Leica Biosystems) and processed by ImageScope. Slides were evaluated independently by two researchers blind to the treatment group.

### Clonogenic assay

The same Large duct-type iCCA used for in vivo experiment was cultured with Metformin 10 µM or without Metformin (Metformin 0 µM, Controls) for 12 and 57 days (pre-treatment). Afterwards, iCCA cells were seeded approximately at the density of 200 cells/well with a fresh medium (without Metformin) and grown for 10 days to form the colonies. Colonies were fixed with 0.1% crystal violet in ethanol (Sigma-Aldrich). The excess dye was removed by washing and colonies were counted. A cluster of at least 50 cells was considered a colony. Then the crystal violet was dissolved, and the absorbance of the extracted dye was measured at 595 nm. The number of colonies was determined and results were expressed as number of colony (absolute number). Quantitative measurement of dimension of the colonies (Colony Dimension Percentage, %) in a single well was calculated as:

$${\text{Colony Dimension }}\% = {\text{Absorbance 595}}\,{\text{nm}}/{\text{Number of colonies}})\cdot{1}00$$

### Statistical analyses

Results are expressed as arithmetic means ± standard deviation (SD). Statistical significance of difference between mean values was assessed using Sigma plot or the analysis of variance when multiple comparisons were performed. A *p* value < 0.05 was considered significant. The Pearson linear correlation index (R) was used to evaluate the association between variables.

## Supplementary Information

Supplementary Information.

## Abbreviations

AMPK: Protein kinase AMP-activated catalytic subunit alpha 2 (PRKAA2)
CSC: Cancer stem cells
Cytokeratin-19: Also known as KRT19 or Keratin 19
E-Cadherin: Also known as CDH1 or Cadherin 1
EMT: Epithelial mesenchymal transition
FOXO3: Forkhead box O3
iCCA: Intrahepatic cholangiocarcinoma
MET: Mesenchymal epithelial transition
mTORC1: Mammalian target of rapamycin
ND: Not detectable
PBGs: Peribiliary glands
PI: Propidium iodide
PSC: Primary sclerosing cholangitis
VIM: Vimentin
WB: Western blot
