# Supplementary material for: Metformin exerts anti-cancerogenic effects and reverses epithelial-to-mesenchymal transition trait in primary human intrahepatic cholangiocarcinoma cells
Source: Sci Rep. 2021 Jan 28;11:2557. doi: 10.1038/s41598-021-81172-0 (PMC7844056; doi:10.1038/s41598-021-81172-0)

Metformin exerts anti-cancerogenic effects and reverses Epithelial-to-Mesenchymal Transition trait in primary human intrahepatic Cholangiocarcinoma cells

Sabina Di Matteo[1,2*], Lorenzo Nevi[2,3], Diletta Overi[4], Nadine Landolina[1], Jessica Faccioli[2], Federico Giulitti[4], Chiara Napoletano[5], Andrea Oddi[6], Augusto M. Marziani[7], Daniele Costantini[2], Agostino M. De Rose[8], Fabio Melandro[9], Maria C. Bragazzi[10], Gian Luca Grazi[6], Pasquale B. Berloco[9], Felice Giuliante[8], Giuseppe Donato [2], Lorenzo Moretta[1], Guido Carpino[11], Vincenzo Cardinale[10], Eugenio Gaudio[4], Domenico Alvaro[2].

AFFILIATIONS:

1. Department of Immunology, Bambino Gesù Children's Hospital, IRCCS, Rome, Italy (current affiliation);
2. Department of Translational and Precision Medicine, Sapienza University of Rome, Rome, Italy;
3. Department of Biosciences, University of Milan, Italy (current affiliation);
4. Department of Anatomical, Histological, Forensic Medicine and Orthopedics Sciences, Sapienza University of Rome, Rome, Italy;
5. Department of Experimental Medicine, Sapienza University of Rome, Rome, Italy;
6. Gastroenterology Unit, Regina Elena National Cancer Institute, Rome, Italy;
7. Department of Information, Electronics and Telecommunications Engineering, Sapienza University of Rome, Rome, Italy;
8. Hepatobiliary Unit, Catholic University of the Sacred Heart School of Medicine, Rome, Italy;
9. Department of General Surgery and Organ Transplantation, Sapienza University of Rome, Rome, Italy;
10. Medical-Surgical and Biotechnologies Sciences, Polo Pontino, Sapienza University of Rome, Rome, Italy;
11. Department of Movement, Human and Health Sciences, Division of Health Sciences, University of Rome "Foro Italico", Rome, Italy;

*Corresponding Authors:
Dr. Sabina Di Matteo, PhD, Department of Internal Medicine and Medical Specialities, University of Rome La Sapienza, Rome, Italy, mail: sabina.dimatteo@opbg.net and dimatteo.sabina@gmail.com

Supplementary Table 1. Results of the Pearson linear correlation test (R) with the transcript levels of AMPK and FOXO3a

	Large bile duct-type iCCA		Small bile duct-type iCCA	
**Gene**	48 hrs	96 hrs	48 hrs	96 hrs
**FOXO3a vs Vimentin**	-0.75	**-0.94***	-0.71	**-0.94***
**FOXO3a vs E-Cadherin**	0.42	**0.99***	0.66	0.08
**FOXO3a vs SNAIL1**	**-0.97***	**-0.97***	-0.85	**-0.99***
**FOXO3a vs TWIST1**	-0.60	**-0.96***	-0.17	**-0.91***
**FOXO3a vs Cytokeratin-19**	**0.97***	**0.99***	0.30	0.89
**AMPK vs Vimentin**	-0.63	**-0.96***	-0.22	-0.85
**AMPK vs SNAIL1**	**-0.97***	**-0.98***	**-0.99***	**-0.99***
**AMPK vs E-Cadherin**	0.72	**0.93***	**0.96***	0.73
**AMPK vs Cytokeratin-19**	0.08	0.89	0.85	**0.96***
**AMPK vs TWIST1**	-0.57	**-0.98***	-0.69	-0.81
**AMPK vs FOXO3A**	0.88	**0.91***	0.84	**0.97***
**SNAIL1 vs Vimentin**	-0.82	-0.89	-0.24	**-0.91***

Supplemental material Table 1 shows the linear correlation test was performed with mRNA levels data obtained at 48 and 96 hrs of treatment with Metformin 0, 10, 100µM by qPCR. The values shown in the table correspond to the Pearson correlation coefficient (R). This value correlated the expression of AMPK or FOXO3A and that of epithelial genes (E-Cadherin, Cytokeratin-19) or mesenchymal and EMT genes (Vimentin, SNAIL1, SNAI2, TWIST) in iCCA cells exposed too Metformin.

The significantly correlated linearly genes are indicated with * symbol ($p<0.01$). Data represent at least 9 independent experiments.

Supplementary Table 2. Sequences of primer pairs used for amplifying the genes of interest (GOI) and the internal reference gene (GAPDH)

**Gene**	***Primers* (5'-3')**
GAPDH	F- 'AGCCACATCGCTCAGACAC'<br>R- 'GCCCAATACGACCAAATCC'
Vimentin	F- 'CTGCCAACCGGAACAATGA'<br>R- 'GTACTCAGTGGACTCCTGCTTT'
Cytokeratin-19	F- 'AGGATGCTGAAGCCTGGTT'<br>R- 'GGTCAGTAACCTCGGACCTG'
E-Caderin	F- 'TCACAGTCACTGACACCAACGA'<br>R- 'GGCACCTGACCCTTGTAGTACGT'
SNAIL1	F- 'ACCCCACATCCTTCTCACTG'<br>R- 'TACAAAAACCCACGCAGACA'
SNAIL2	F- 'CTTTTTCTTGCCCTCACTGC'<br>R- 'ACAGCAGCCAGATTCCTCAT'
TWIST1	F- 'ACCATCCTCACACCTCTGCATTCT'<br>R- 'TGCAGGCCAGTTTGATCCCAGTAT'
AMPK	F- 'GACTGCTACTCCACAGAGATCG'<br>R- 'TCAGCATCTGAATCACTCCTTT'
FOXO3a	F- 'CTTCAAGGATAAGGGCGACA'<br>R- 'CGACTATGCAGTGACAGGTTG'

Primers were designed by the PROBEFINDER software (https://www.roche-applied-science.com/sis/rtpcr/upl/index.jsp) and Primer-BLAST software (https://www.ncbi.nlm.nih.gov/tools/primer-blast/).

Supplementary Figure 1A. ***In vitro*** **clonogenic assay of Large duct-type iCCA pre-treated with Metformin 10µM for 12 and 57 days**

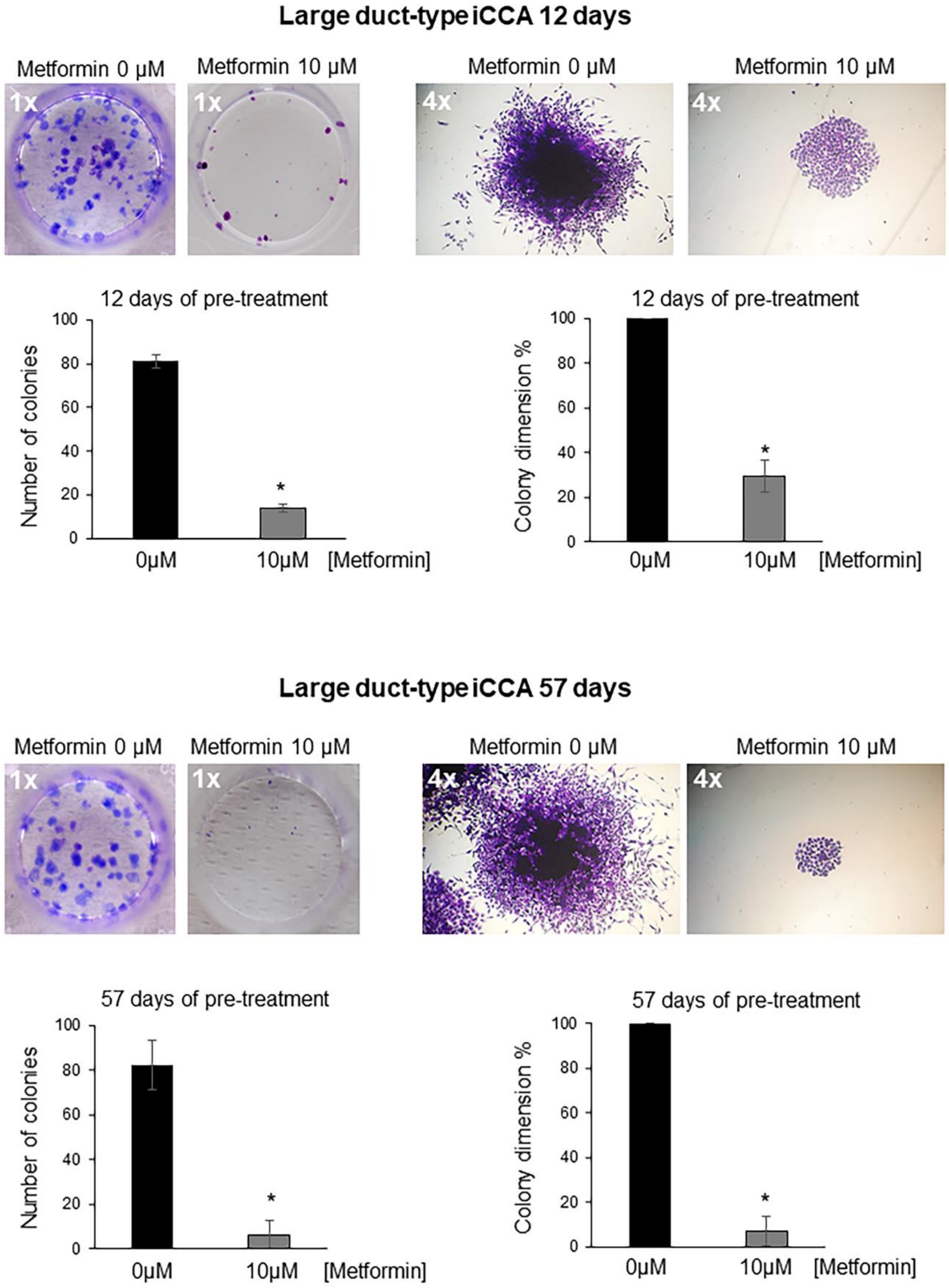

Supplementary Figure 1B. ***In vitro*** **clonogenic assay of Small duct-type iCCA pre-treated with Metformin 10μM for 12 and 57 days**

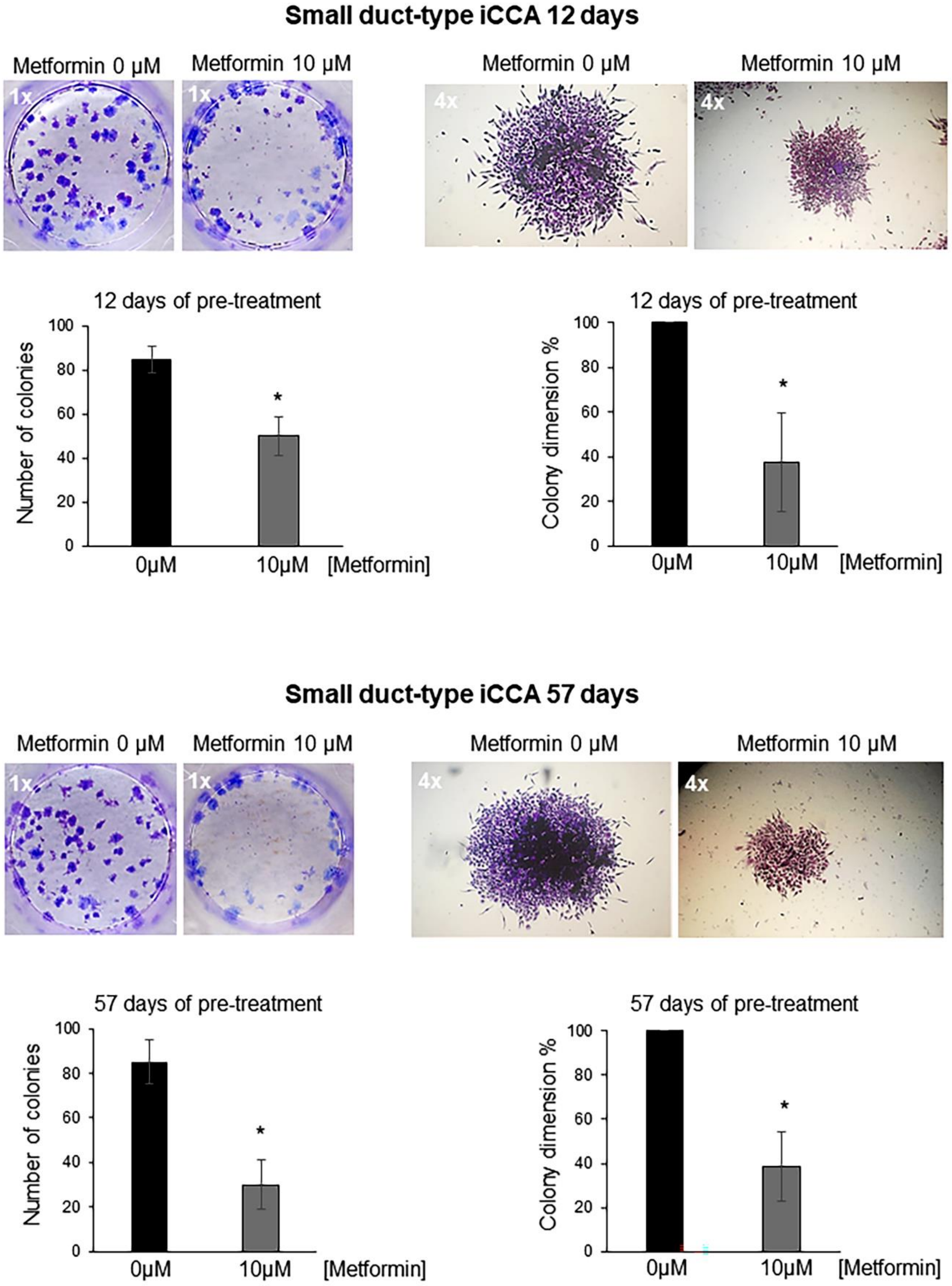

After 12 or 57 days of chronic treatment with Metformin 10μM or without Metformin (Metformin 0μM, Controls), cells were seeded (150 cells/well) and after further 10 days of culture (without Metformin), Large duct-type iCCA colonies were stained with 0.1% crystal violet.

Photographs (magnification 1x): show colonies formed by Large duct-type iCCA (A) and Small duct-type iCCA (B) with visible decrease in the number of colonies in pre-treated cells. Bar chart

of number of colonies shows a significant reduction in the number of colonies pre-treated with Metformin 10µM compared to controls. A colony was considered a cluster of at least 50 cells. Data represent mean ± SD of N=5 independent experiments. * $p<0.05$ vs controls.

Photographs (magnification 4x): show representative colonies formed by Large duct-type iCCA (A) and Small duct-type iCCA (B), with evident decreased dimension (colony dimension %). In Large duct-type iCCA, a dramatic change in the cells phenotype was observed upon pre-treatment with Metformin 10µM. The bar chat shows the decrease of colony dimension %. * $p<0.05$ vs Controls. Data represent mean ± SD of N=5 independent experiments.

Supplementary Figure 2. Scheme

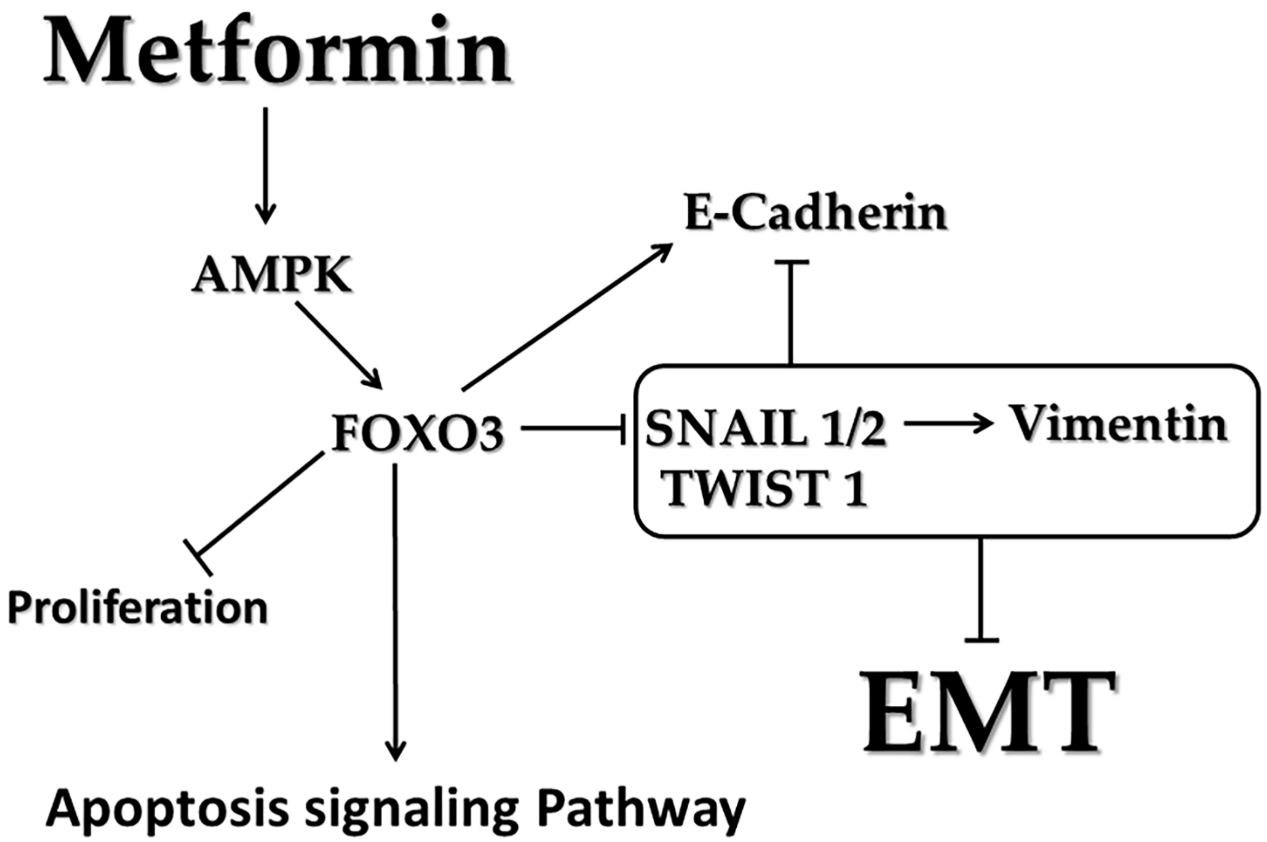

Metformin triggered AMPK and FOXO3 gene expression; these could represent the molecular mechanisms at the basis of the cell proliferation blockage, the induction of apoptosis, and the reversion of EMT.

Supplementary Figure 3. Phase contrast microscopy images and histology of iCCA

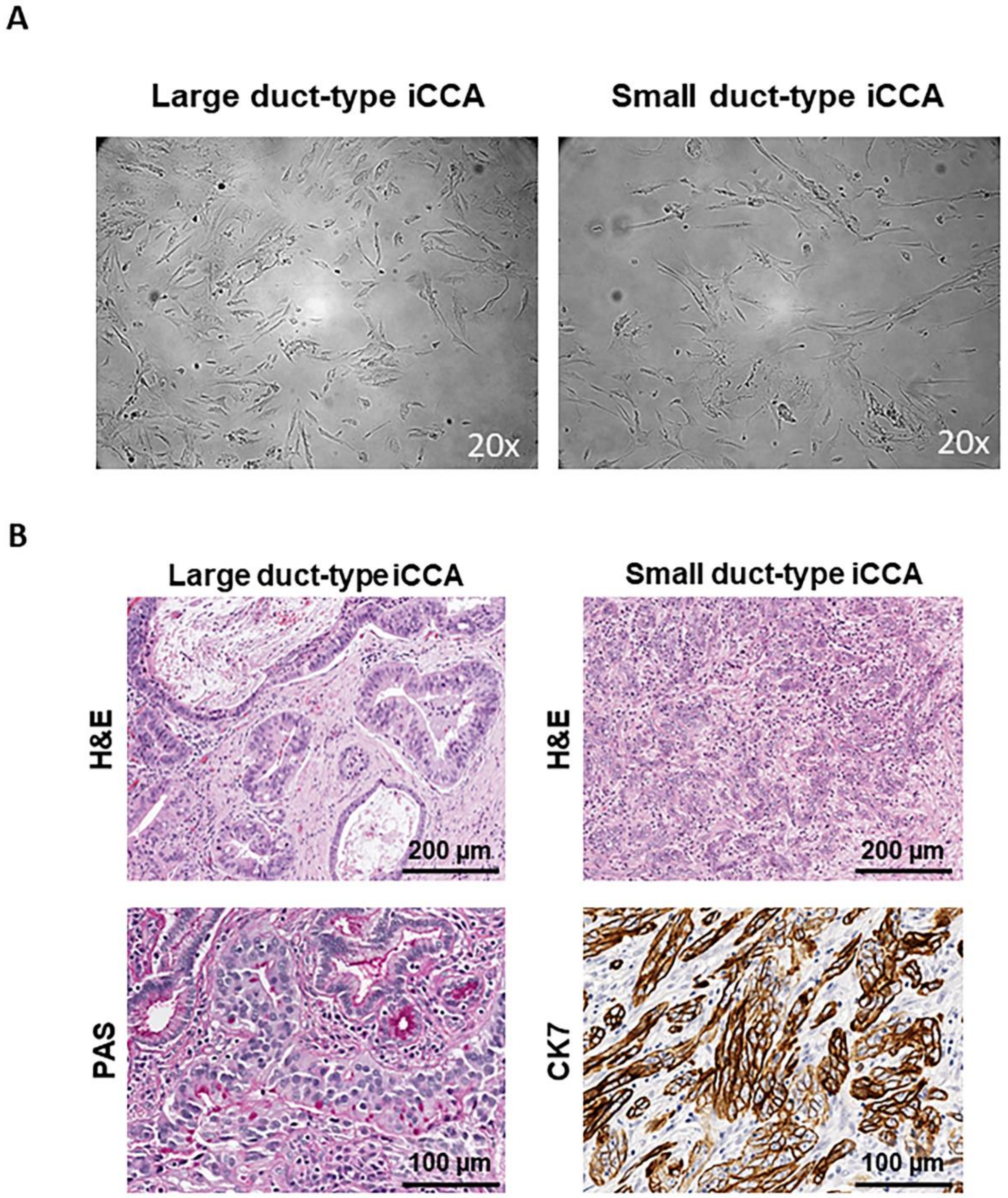

A. Phase contrast microscopy images (magnification 20x), representative images of cells in the slides used for the characterization of the cells in figure 1A.

B. Representative images of surgical tumour specimens from Large duct-type iCCA (left panels) and Small duct-type iCCA (right panels). Large duct-type iCCAs were composed of columnar, mucin-producing (PAS+) tumour cells lining large neoplastic ductular or papillary structures. Small duct-type iCCAs were constituted of small-sized neoplastic ducts with no or minimal mucin production; H&E: haematoxylin and eosin; CK7: Cytokeratin-7; PAS: periodic acid-Schiff.

Supplementary Figure 4. Mice Large duct-type iCCA cells xenografts

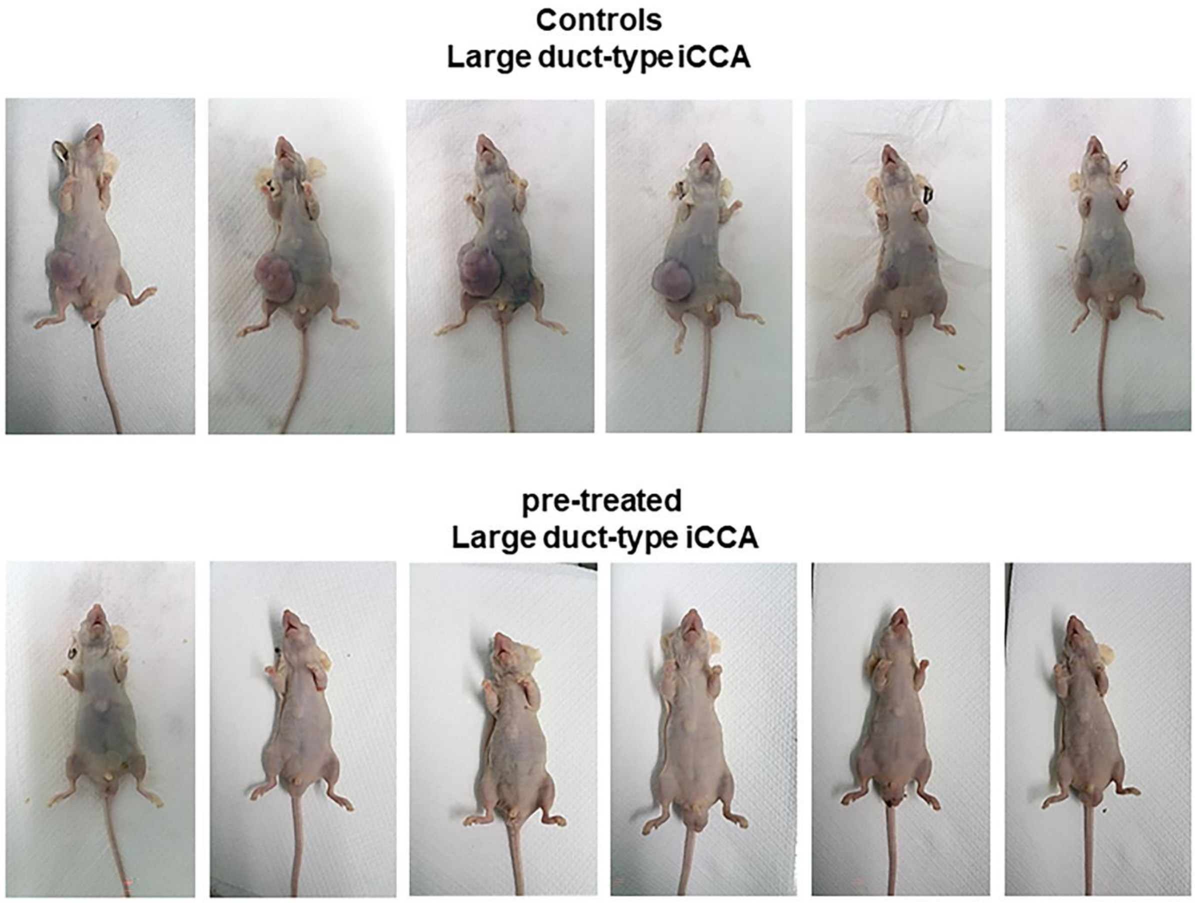

Images (magnification 1x) of mice tumour xenografts on the day of sacrifice. Large duct-type iCCA cells were pre-treated with Metformin 0µM (Controls) or Metformin 10µM for 57 days and then injected subcutaneously. After 10 weeks a tumour mass into flank of mice was visible whereas in the mice injected with the cells pre-treated with Metformin 10µM, no tumour mass is macroscopically visible.

Supplementary Figure 5. Full-length membranes (western blot)

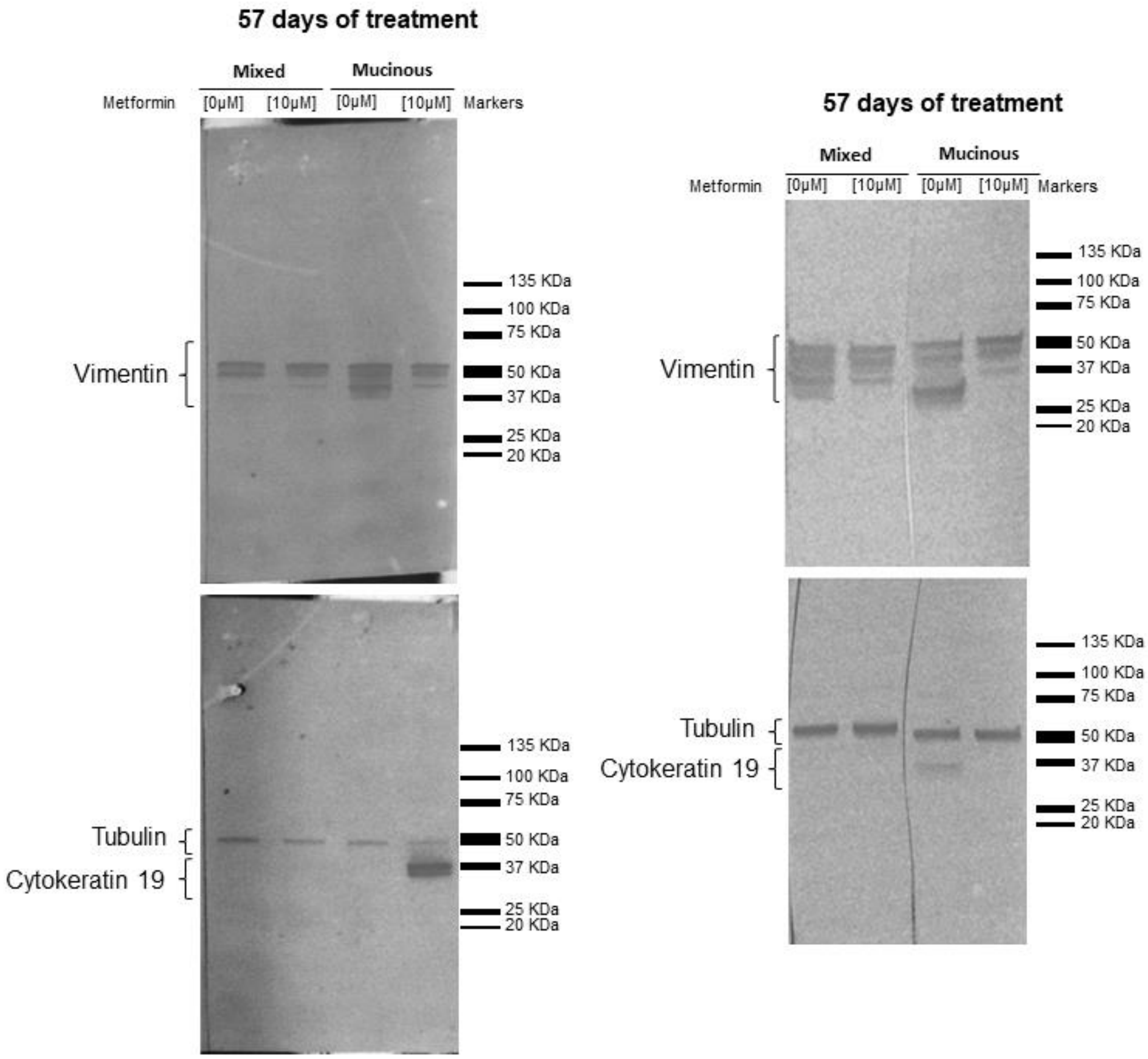

Supplement: Supplementary file 1 — Supplementary Information. [file 41598_2021_81172_MOESM1_ESM.pdf]
